# Advances in clinical applications of cardiovascular magnetic resonance imaging

**DOI:** 10.1136/hrt.2007.119016

**Published:** 2008-01-20

**Authors:** W P Bandettini, A E Arai

**Affiliations:** Laboratory of Cardiac Energetics, National Heart, Lung, and Blood Institute, National Institutes of Health, Bethesda, Maryland, USA

## Abstract

Cardiovascular magnetic resonance (CMR) is an evolving technology with growing indications within the clinical cardiology setting. This review article summarises the current clinical applications of CMR. The focus is on the use of CMR in the diagnosis of coronary artery disease with summaries of validation literature in CMR viability, myocardial perfusion, and dobutamine CMR. Practical uses of CMR in non-coronary diseases are also discussed.

Box 1 Appropriate indications for the use of CMR^142^*

Detection of CAD: Symptomatic—evaluation of chest pain syndrome (use of vasodilator perfusion CMR or dobutamine stress function CMR)

- Intermediate pre-test probability of CAD
- ECG uninterpretable OR unable to exercise

Detection of CAD: Symptomatic—evaluation of intracardiac structures (use of MR coronary angiography)

- Evaluation of suspected coronary anomalies

Risk assessment with prior test results (use of vasodilator perfusion CMR or dobutamine stress function CMR)

- Coronary angiography (catheterisation or CT)
- Stenosis of unclear significance

Structure and Function—evaluation of ventricular and valvular function

Procedures may include LV/RV mass and volumes, MR angiography, quantification of valvular disease, and delayed contrast enhancement

- Assessment of complex congenital heart disease including anomalies of coronary circulation, great vessels, and cardiac chambers and valves
- Procedures may include LV/RV mass and volumes, MR angiography, quantification of valvular disease, and contrast enhancement
- Evaluation of LV function following myocardial infarction OR in heart failure patients
- Patients with technically limited images from echocardiogram
- Quantification of LV function
- Discordant information that is clinically significant from prior tests
- Evaluation of specific cardiomyopathies (infiltrative (amyloid, sarcoid), HCM, or due to cardiotoxic therapies)
- Use of delayed enhancement
- Characterisation of native and prosthetic cardiac valves—including planimetry of stenotic disease and quantification of regurgitant disease
- Patients with technically limited images from echocardiogram or TEE
- Evaluation for arrhythmogenic right ventricular cardiomyopathy (ARVC)
- Patients presenting with syncope or ventricular arrhythmia
- Evaluation of myocarditis or myocardial infarction with normal coronary arteries
- Positive cardiac enzymes without obstructive atherosclerosis on angiography

Structure and Function—evaluation of intracardiac and extracardiac structures

- Evaluation of cardiac mass (suspected tumour or thrombus)
- Use of contrast for perfusion and enhancement
- Evaluation of pericardial conditions (pericardial mass, constrictive pericarditis)
- Evaluation for aortic dissection
- Evaluation of pulmonary veins prior to radiofrequency ablation for atrial fibrillation
- Left atrial and pulmonary venous anatomy including dimensions of veins for mapping purposes

Detection of myocardial scar and viability—evaluation of myocardial scar (use of late gadolinium enhancement)

- To determine the location and extent of myocardial necrosis including “no reflow” regions
- Post acute myocardial infarction
- To determine viability prior to revascularisation
- Establish likelihood of recovery of function with revascularisation (PCI or CABG) or medical therapy
- To determine viability prior to revascularisation
- Viability assessment by SPECT or dobutamine echo has provided “equivocal or indeterminate” results

*adapted from ACCF/ACR/SCCT/SCMR/ASNC/NASCI/SCAI/SIR 2006 appropriateness criteria for cardiac computed tomography and cardiac magnetic resonance imaging. *J Am Coll Cardiol* 2006;**48**:1475–97.

The purpose of this review is to illustrate that cardiovascular magnetic resonance (CMR) has developed into a powerful non-invasive diagnostic tool that can routinely image myocardial anatomy, function, perfusion, and viability without need for ionising radiation.

## BASIC HARDWARE

Fundamentally, CMR uses a magnet 30 000 to 60 000 times the strength of the Earth’s magnetic field to detect the location and physical properties of protons in the body. CMR requires fast gradients, phased-array coils, cardiac gating, and cardiovascular software. Higher magnet field strength (3T vs 1.5T) improves signal-to-noise but exacerbates problems related to field inhomogeneity and specific absorption of radiation, factors leading to artifacts and patient heating respectively. The gradients encode many aspects of the image including position in the body, velocity of blood, and other parameters. Phased-array coils act as antennae to receive the tiny MRI-related radiofrequency signals emanating from the body. Phased-array coils enable image acquisition acceleration with parallel imaging methods.^1^^–^3

Stress testing requires MRI-compatible intravenous pumps, contrast injectors, patient monitoring equipment, resuscitation equipment, and audiovisual equipment to communicate with the patient. The clinical team must be prepared to quickly remove a patient from the scanner and treat cardiovascular emergencies.

## CONTRAINDICATIONS

The magnetic fields, gradients, and radiofrequency pulses used in MRI pose risks to patients and staff, requiring meticulous safety procedures. Ferromagnetic materials should not be taken into the scanner room. Neurovascular clips, pacemakers, automatic implantable defibrillators, cochlear implants, metal in the eye, retained shrapnel, and neurostimulators are contraindications to MRI although certain models may be safe. With CMR imaging, it is important to note that intracoronary stents and coronary artery bypass graft surgery are not contraindications.^4^ Although small forces are generated within metal heart valves by the magnetic fields, they are minimal compared with the forces generated by the beating heart, and all mechanical heart valves are considered safe. When in doubt, various resources, such as www.imrser.org and www.mrisafety.com,^5^ are available to check a device’s safety within an MRI scanner.^6^^–^9

## WHAT CMR CAN DO

### Assessment of right and left ventricular function and mass

Assessment of left ventricular size, function and mass has been well validated in both autopsy and animal studies,^10^^–^12 and has excellent intraobserver and interobserver variability.^13^^–^18 This reproducibility allows for smaller sample size in studies requiring serial exams than other lower-resolution imaging such as echocardiography.

CMR can quantify regional wall motion and myocardial strain with techniques such as the harmonic phase method (HARP),^19^ displacement encoding with stimulated echoes (DENSE),^20^ ^21^ and spatial modulation magnetisation (SPAMM).^22^ These techniques can assess myocardial strain independent of the effects of through-plane motion.

Real-time CMR can be used in situations where cardiac gating is not currently feasible. One example is the prenatal assessment of fetal cardiovascular abnormalities.^23^

### Diagnosis of coronary artery disease

**Figure 1:**
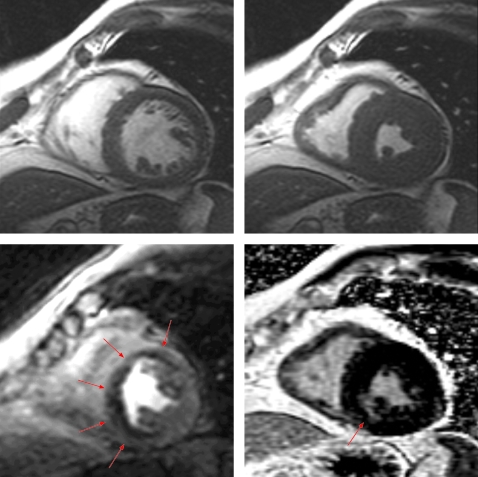
Comprehensive cardiovascular magnetic resonance with cine function, dipyridamole perfusion, and delayed enhancement: A 77-year-old man presents with exertional angina and a past medical history significant for hypertension and a prior stroke. In the top row, cine function demonstrates normal global and regional left ventricular systolic function. The dipyridamole perfusion image on the lower left panel demonstrates a severe perfusion defect in a multivessel coronary distribution, while the delayed enhancement image on the right lower panel demonstrates only a small subendocardial myocardial infarction of the inferoseptal wall, indicating a large ischaemic region with a large territory of viable myocardium.

A single CMR study can provide information regarding the coronary arteries, left ventricular systolic function, myocardial perfusion, and viability (fig 1).

#### Viability assessment

**Figure 2:**
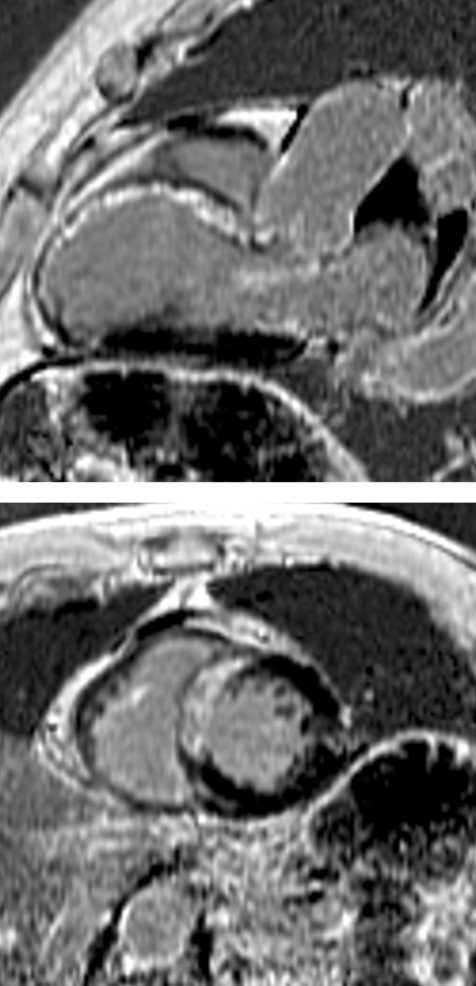
Delayed enhancement in a patient with a near-transmural anteroseptal myocardial infarction.

One of the major breakthroughs for the use of CMR was the development of gadolinium delayed enhancement techniques to assess for myocardial infarction.^24^ Gadolinium shortens tissue T1 relaxation time, a magnetic property inherent to all tissues. The operator can select an inversion time that will “null” normal myocardium resulting in images where viable myocardium appears uniformly dark while a region of myocardial infarction or fibrotic scar appears bright (fig 2). Dysfunctional but viable myocardium is expected to have functional recovery if revascularised (in the case of hibernating myocardium), with time (in the case of stunned myocardium), or with resynchronisation (in the case of dyssynchronous myocardium).

In a seminal paper by Kim *et al*, the delayed enhancement of myocardial infarction by CMR closely correlated with the histopathological triphenyltetrazolium chloride (TTC) findings.^25^ Multiple studies have demonstrated the inverse relationship between the transmural extent of myocardial infarction and recovery of function, the higher spatial resolution of this technique compared with nuclear techniques, as well as the good correlation with biomarkers of necrosis.^26^^–^48 The reproducible nature of the delayed enhancement technique also makes it a natural choice for serial imaging of chronic infarctions.^40^

#### Myocardial perfusion

Myocardial perfusion has been a CMR research focus. The challenge has been obtaining enough signal, temporal resolution, spatial resolution, and spatial coverage, while minimising artifacts. Most groups use fast gradient recalled echo (FGRE), FGRE with echoplanar imaging (Hybrid EPI), and steady state free precession (SSFP) perfusion techniques, typically using adenosine or dipyridamole as the stressor. These sequences may be accelerated with parallel imaging techniques and performed with multiple gadolinium dosing schemes. The studies may be interpreted qualitatively, semi-quantitatively, or quantitatively. Despite the technical issues related to perfusion imaging, many papers document that CMR first-pass perfusion has comparable diagnostic accuracy to the alternative myocardial perfusion imaging standards.^49^^–^70

#### Dobutamine CMR

Dobutamine stress CMR was first described in the same year that dobutamine stress echocardiography was described.^71^ Dobutamine CMR has good sensitivity and specificity in the detection of significant coronary artery disease (table 1) with a safety profile similar to dobutamine echocardiography.^72^ While the sensitivity and specificity of CMR are comparable to stress echocardiography in patients with good echocardiographic windows, CMR performs better than stress echocardiography in patients with suboptimal echocardiographic windows.^73^^–^78 Furthermore, dobutamine stress CMR has prognostic value above and beyond the baseline ejection fraction.^79^ ^80^

**Table 1:** Summary of dobutamine validations

Year	First Author	N	Excluded	Reference	Sensitivity	Specificity
2006	Paetsch^77^	150	0	Cath >50%	78	87
2006	Jahnke^75^	40	0	Cath >50%	82	87
2004	Paetsch^62^	79		Cath >50%	89	80
2004	Wahl^78^	170	10	Cath >50%	89	84
1999	Hundley^74^	163	10	Cath >50%	83	83
1999	Nagel^76^	208	36	Cath >50%	86	86

#### Acute chest pain in the hospital setting

Three major papers have looked at use of CMR in patients with acute coronary syndrome (ACS) or early diagnosis of chest pain in the emergency department. In a study of 161 patients presenting with chest pain not associated with ST elevation, Kwong *et al* found that CMR had 100% sensitivity for non-ST elevation myocardial infarction and was a better predictor of ACS than standard clinical tests including the composite TIMI risk score.^81^ In a higher risk group of 68 patients with possible or probable ACS scheduled for coronary angiography, Plein *et al* found that a multi-component CMR consisting of cine function, adenosine and rest perfusion, delayed enhancement, and coronary artery imaging yielded a sensitivity of 96% and a specificity of 83% in predicting the presence of significant coronary artery disease.^64^ In another emergency department study of 141 patients with myocardial infarction excluded by serial troponin assays, Ingkanisorn *et al* found that adenosine stress CMR had excellent prognostic value as 100% of patients with adverse cardiovascular outcomes were detected with an overall specificity of 91%.^54^

CMR is also helpful in patients with atypical chest pain.^82^ For example, many patients with myocarditis present with chest pain, ECG abnormalities, elevated biomarkers, but normal coronary arteries. This diagnosis is easily made with CMR. The presence of atypical mid-wall or epicardial delayed enhancement distinguishes myocarditis from MI.^83^ ^85^ Stress CMR perfusion can detect diffuse subendocardial ischaemia in patients with syndrome X.^86^ Acute chest pain from acute aortitis will present with irregularly thickened aortic wall and bright enhancement of the aortic wall on delayed enhancement imaging.^87^ ^88^ CMR has been used in the diagnosis of stress cardiomyopathy (tako tsubo, left ventricular apical ballooning syndrome, and broken heart syndrome). Despite the profound left ventricular apical systolic dysfunction, there is little delayed enhancement in these patients.^89^^–^92

#### Coronary artery imaging

Although multidetector computed tomography (MSCT) is the most rapid and highest-resolution non-invasive technique for imaging the coronary arteries, CMR offers an alternative for imaging the coronary arteries. CMR does not require ionising radiation and can be combined with a multimodality CMR assessment of cardiac function, perfusion, and viability in a relatively short period of time.^93^ However, coronary imaging by CMR is still relatively complicated and many technical nuances require significant operator experience.

**Figure 3:**
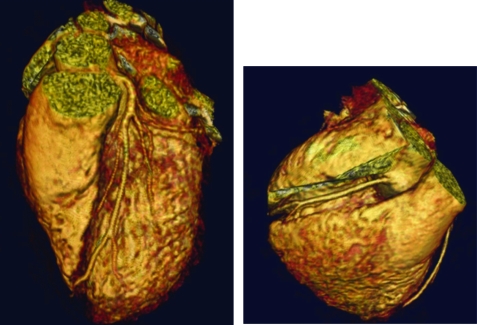
Whole heart coronary magnetic resonance angiography. Image provided courtesy of Vibhas Deshpande, MR Research & Development, Siemens Medical Solutions.

A few studies indicate that CMR is not as far from clinical feasibility as many physicians assume. A multicentre study of 109 patients who underwent coronary magnetic resonance angiography (MRA) reported a sensitivity of 100%, a specificity of 85%, and an accuracy of 87% in the detection of left main artery or three-vessel disease.^94^ Sakuma *et al* performed three-dimensional whole-heart coronary MRA in 131 patients with a mean acquisition time of 12.9 (SD 4.3) minutes and a per patient sensitivity of 82%, specificity of 90%, and accuracy of 87%.^95^ However, most experts and clinical guidelines only support the use of CMR in determining the proximal course of anomalous coronary arteries (fig 3, coronary MRA).

### Cardiomyopathy

CMR can characterise cardiomyopathies in unique ways based on the magnetic properties of myocardium.^96^^–^99 Assomull *et al* succinctly review the use of CMR in the evaluation of congestive heart failure.^100^

In hypertrophic cardiomyopathy, CMR can detect patches of myocardial fibrosis with intermediate delayed enhancement.^101^^–^103 CMR can diagnose hypertrophy missed by echocardiography and more accurately determine the extent of hypertrophy.^104^

In patients suspected of having arrhythmogenic right ventricular dysplasia/cardiomyopathy (ARVD/C), CMR can detect global right ventricular abnormalities, right ventricular aneurysms, or regional wall motion abnormalities. Fibrofatty myocardial infiltration can be determined in patients suspected of having ARVD/C.^105^ Sen-Chowdhry *et al* have proposed modified criteria for the diagnosis of ARVD/C focusing on right ventricular size and function, right ventricular segmental dilatation, and regional right ventricular hypokinesis. These proposed criteria would improve the sensitivity in the detection of early or incompletely expressed disease.^106^

CMR can measure iron overload in the heart, particularly as a result of thalassaemia.^73^ ^107^ Iron overload shortens T2* relaxation properties of the myocardium and liver. Intriguingly, some patients with thalassaemia have iron overload in the heart but not in the liver and vice versa.^73^ Thus, CMR determinations of iron overload may be better at assessing patient risk than relying on liver biopsy alone and may be used to follow therapy success.

CMR is good at differentiating constrictive from restrictive cardiomyopathy due to each entity’s unique presentation and physiology. Many of the infiltrative cardiomyopathies such as amyloidosis, sarcoidosis, Chagas’ disease, and endomyocardial fibroelastosis have characteristic abnormalities on delayed enhancement.^97^ ^99^ ^108^^–^112 CMR can identify thickened pericardium as well as abnormal motion of the heart in constrictive cardiomyopathy. While both CT and CMR can detect thickened pericardium, CMR is better able to distinguish between pericardial thickening and small effusion than CT.^113^ Real-time imaging to evaluate the septum may demonstrate interventricular dependence.^114^ Real-time cine imaging of the inferior vena cava during respiration can also separate constrictive from restrictive physiology.^115^

### Congenital heart disease

In a patient with congenital heart disease, anatomic connections or malformations may be identified, the direction of intracardiac shunts may be identified and quantified, and valvular anatomy and function may be assessed. Volumetric anatomic CMR depicts the complex vascular abnormalities associated with congenital syndromes and the surgical corrections. Echocardiography cannot always visualise the heart and great vessels in their entirety, particularly in adults with surgically corrected congenital heart disease. Repeated exposure to the radiation of CT is not desirable, especially in a paediatric population that is at greater risk for developing long-term radiation-related malignancies.^116^

**Figure 4:**
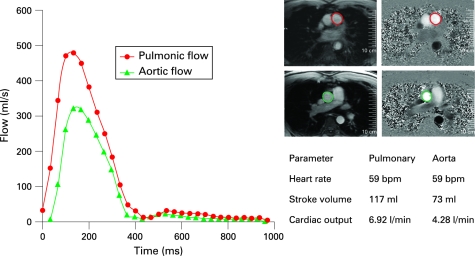
Pulmonic flow (Qp) and systemic flow (Qs) may be calculated non-invasively with cardiovascular magnetic resonance using simple phase-contrast techniques. This figure illustrates an abnormal Qp:QS of 1.6:1 in a patient with an atrial septal defect.

CMR can provide more than simply anatomical imaging. A saturated black band technique highlights intracardiac shunting. Velocity encoded phase contrast techniques can quantify the severity of intracardiac shunts. Measuring pulmonary blood flow (Qp) in the pulmonary artery and systemic blood flow (Qs) in the aorta provides a noninvasive estimate of Qp/Qs and thus quantifies the degree of intracardiac shunting (fig 4). CMR can quantify the amount of valvular regurgitation (eg, in patients with Tetralogy of Fallot).

### Valvular disease

**Figure 5:**
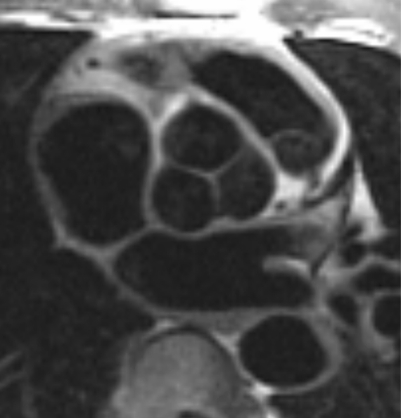
Black-blood fast spin echo technique to visualise the aortic valve.

**Figure 6:**
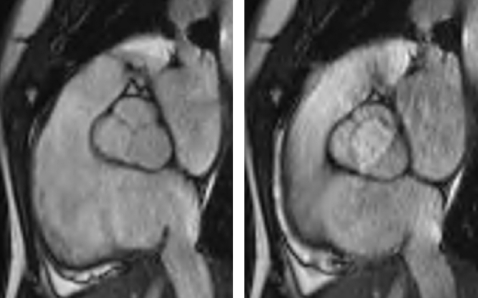
During diastole cine imaging, an aortic valve appears tricuspid; however, during systole, it is apparent that the valve is functionally bicuspid with fusion of the right and left cusps.

CMR provides non-invasive clear anatomical valvular information that can impact clinical management of a patient. It is possible to differentiate a bicuspid from a tricuspid aortic valve (figs 5 and 6). CMR reproducibly characterises aortic valve anatomy and the determined aortic valve area correlates well with cardiac catheterisation.^117^

Phase contrast techniques can reliably measure peak velocity and thus peak gradient in aortic stenosis. Valvular information in combination with accurate left ventricular volumes and assessment of thoracic aortic dilatation can assist in planning valvular replacement and, importantly, determine whether the aorta needs intervention as well. Similar data can be obtained in an assessment of the pulmonic valve, which is not always well-defined by transthoracic echocardiography.

While most valvular lesions seen by echocardiography can be assessed by CMR, echocardiography has the advantages of widespread availability and validation. CMR provides additional information in patients who have poor echocardiographic windows and is useful in patients who are poor candidates for invasive transoesophageal echocardiography or when additional surgery beyond the valve is contemplated.

### Assessment of cardiac masses

**Figure 7:**
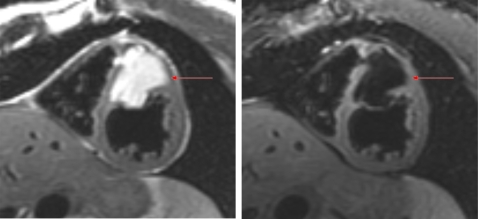
A 48-year-old woman presented with a markedly abnormal preoperative ECG and nuclear stress test indicating that she had an anteroseptal myocardial infarction. Cardiovascular magnetic resonance was able to demonstrate that the patient actually had an intraseptal mass (bright on the left) which was in fact a benign lipoma as demonstrated by fat saturation techniques (dark on the right after using a fat saturation technique to suppress the fat).

Through various tissue-characterising techniques (T2-weighted, T1-weighted, first-pass perfusion, and delayed enhancement), CMR can reliably distinguish between myocardium, fat, avascular tissue (eg, thrombus), and other tissue types, such as tumours (fig 7). CMR often aids in differentiating intracardiac masses from masses that externally compress the heart.

The ability to characterise normal structures or variants makes CMR superior to echocardiography in the assessment of intracardiac mass. Atrial structures such as Eustachian valve, crista terminalis, Chiari network, and lipomatous hypertrophy are commonly mistaken by echocardiography to be a mass, and CMR can help avoid more invasive diagnostic testing.^118^ Contrast-enhanced CMR is twice as sensitive as echocardiography in the detection of ventricular thrombi.^119^^–^121

### Non-coronary vascular imaging

#### Aorta and great vessels

MRI and MRA can assess large and medium-sized vascular structures. Serial exams are particularly useful in the paediatric population with congenital abnormalities of the aorta. CMR is able to visualise congenital aortic abnormalities including right-sided aortic arch, cervical aortic arch, double aortic arch, and vascular ring. As many as 42% of surgically repaired coarctations present with restenosis, dissection, pseudoaneurysm, or aneurysm at a later date.^122^^–^124

**Figure 8:**
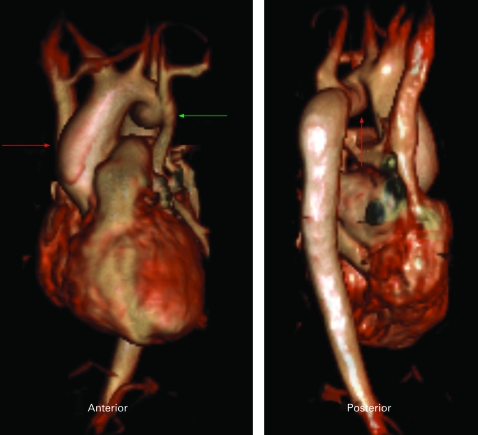
This magnetic resonance angiography was performed in a Turner’s Syndrome patient. Note on the anterior view the dilated size of the ascending aorta (red arrow) in comparison with the descending aorta, as well as the persistent left-sided superior vena cava (green arrow). The posterior view demonstrates the malformed aortic arch (red arrow).

Other common indications for CMR include assessment of aortic dilation and aneurysm, aortic dissection, aortic ulcer, and intramural haematoma. While a contrast CT is the study of choice in the acutely ill, haemodynamically unstable patient, in a haemodynamically stable patient a focused CMR exam of the aorta may be performed within approximately 10–15 minutes with little cooperation from the patient (fig 8). CMR is more sensitive than CT, echocardiography, and transoesophageal echocardiography in the diagnosis of intramural haematoma. CMR can also distinguish between an acute intramural haematoma and a chronic haematoma based upon the T1 and T2 characteristics of the bleed.^125^

#### Pulmonary veins

Three-dimensional MRA can help guide electrophysiological interventions and can detect pulmonary vein stenosis after the procedure. It is possible to merge 3D MRA with fluoroscopy in the electrophysiology lab to help guide catheter tip placement and the ablation. CMR is also useful for determining the flow patterns through vessels.^126^

## FUTURE DIRECTIONS

CMR continues to develop rapidly. Contrast agents targeted to specific tissue types are in development. For example, thrombus-avid contrast agents are feasible.^127^^–^129 Lipid-specific agents have also been studied. Stem cells and macrophages have been identified with iron-based contrast agents and tracked in vivo.^130^^–^133

Interventional CMR is also a field with growing interest. A variety of percutaneous procedures used to treat vascular abnormalities and congenital heart disease are in development.^134^^–^137 Even CMR-guided percutaneous replacement of the aortic valve is feasible.^138^ CMR can help precisely guide delivery of drugs and stem cells.^139^^–^141

## LIMITATIONS

There are many factors that have slowed the dissemination of CMR. CMR is expensive and requires a skilled multidisciplinary team. In-depth CMR training is not readily available. Insufficient numbers of adequately trained physicians limit utilisation and dissemination of CMR. In many countries, reimbursement of CMR is not well-established. Although gadolinium-based contrast agents are in everyday clinical use worldwide, cardiovascular applications are not yet approved by the United States Food and Drug Administration. Currently it is easier to run an MRI for profit by doing non-cardiac applications. Thus, significant economic issues must be addressed.

MRI scanners trigger claustrophobia in many patients. Other patients cannot undergo MRI scans due to implanted devices like pacemakers or defibrillators. Arrhythmias and respiratory insufficiency compromise many of the highest quality CMR methods. Technology development can solve most of these issues.

## CONCLUSION

With advances in CMR technology, multiple clinical indications have followed. Although there is overlap with other cardiac imaging modalities, CMR often works in a complementary fashion to these other techniques or resolves residual diagnostic dilemmas. The strengths of CMR lie in its ability to comprehensively image cardiac anatomy, function, perfusion, viability and physiology, and put this information in the context of the wide field of view of surrounding vascular and non-cardiac anatomy. At a time when serious concerns are being raised about the medical use of ionising radiation, it is reassuring to know that CMR provides high-quality diagnostic information without a need for radiation.

**Table 2:** Summary of gadolinium delayed enhancement publications

Year	Authors	n	Acute vs chronic	Major findings
2006	Baks T *et al*^27^	27	Acute	Delayed enhancement predicted recovery of function.
			Chronic	
2006	Gerber BL *et al*^31^	16	Acute	Delayed enhancement correlated with MI size.
		21	Chronic	
2005	Baks T *et al*^26^	22	Acute	Delayed enhancement predicted recovery of function.
			Chronic	
2005	Bello D *et al.*^29^	48	Chronic	Delayed enhancement correlated with MI size and predicted inducibility of ventricular tachycardia.
2005	Ibrahim T *et al*^33^	33	Acute	Delayed enhancement correlated with MI size.
2005	Selvanayagam JB *et al*^45^	50	Acute	Delayed enhancement correlated with biomarkers of necrosis.
		24	Chronic	
2004	Ingkanisorn WP *et al*^34^	33	Acute	Delayed enhancement predicted recovery of function and correlated with biomarkers of necrosis.
		20	Chronic	
2004	Lund GK *et al*^39^	60	Acute	Delayed enhancement correlated with MI size.
2004	Nelson C *et al*^41^	60	Chronic	Delayed enhancement predicted recovery of function.
2004	Selvanayagam JB *et al*^44^	52	Chronic	Delayed enhancement predicted recovery of function.
2004	Wellnhofer E *et al*^47^	29	Chronic	Delayed enhancement and dobutamine CMR predicted recovery of function.
2003	Beek AM *et al*^28^	30	Acute	Delayed enhancement predicted recovery of function.
			Chronic	
2003	Knuesel PR *et al*^37^	19	Chronic	Delayed enhancement predicted recovery of function.
2003	Kühl HP *et al*^38^	26	Chronic	Delayed enhancement correlated with MI size.
2003	Wagner A *et al*^46^	91	Chronic	Delayed enhancement correlated with MI size.
2002	Gerber BL *et al*^32^	20	Acute	Delayed enhancement predicted recovery of function.
			Chronic	
2002	Klein C *et al*^36^	31	Chronic	Delayed enhancement correlated with MI size.
2002	Mahrholdt H *et al*^40^	20	Chronic	Delayed enhancement correlated with MI size and was reproducible in two separate scans.
2002	Perin EC *et al*^42^	15	Chronic	The unipolar voltage recorded during electromechanical mapping varied inversely with the amount of delayed enhancement.
2001	Choi KM *et al*^30^	24	Acute	Delayed enhancement predicted recovery of function and correlated with biomarkers of necrosis.
			Chronic	
2001	Ricciardi MJ *et al*^43^	14	Acute	Delayed enhancement correlated with biomarkers of necrosis. Microinfarcts were detected in patients who had PCI-related elevations in CKMB.
		6	Chronic	
2001	Wu E *et al*^48^	82	Chronic	Delayed enhancement correlated with MI size.
2000	Kim RJ *et al*^35^	50	Chronic	Delayed enhancement predicted recovery of function.

CKMB, muscle and brain subunits of creatine kinase; CMR, cardiovascular magnetic resonance; MI, myocardial infarction; PCI, percutaneous coronary intervention.

**Table 3:** Summary of vasodilator perfusion CMR validation publications

Year	First author	n	Excluded	Stress	Reference	Sensitivity	Specificity
2007	Merkle *et al*^70^	228	0	Adenosine	Cath >50%	93	86
2006	Ingkanisorn *et al*^54^	141	4	Adenosine	Prognosis	100	93
2006	Klem *et al*^58^	92	3	Adenosine	Cath >70%	89	87
2006	Pilz *et al*^63^	176	5	Adenosine	Cath >70%	96	83
2006	Rieber *et al*^66^	50	7	Adenosine	Cath >50% and FFR	88	90
2005	Okuda *et al*^60^	33	0	Dipyridamole	Cath >70%	84	87
2005	Plein *et al*^65^	92		Adenosine	Cath >70%	88	82
2005	Sakuma *et al*^67^	40	0	Dipyridamole	Cath >70%	81	68
2004	Bunce *et al*^50^	35	0	Adenosine	Cath >50%	74	71
2004	Giang *et al*^52^	94	14	Adenosine	Cath >50%	93	75
2004	Kawase *et al*^56^	50	0	Nicorandil	Cath >70%	94	94
2004	Paetsch *et al*^61^	49	0	Adenosine	Cath >75%	89	80
2004	Paetsch *et al*^62^	79		Adenosine	QCA >50%	91	62
2004	Plein *et al*^64^	72	4	Adenosine	Cath >70%	88	83
2004	Takase *et al*^69^	102	0	Dipyridamole	Cath >50%	93	85
2003	Doyle *et al*^51^	199	15	Dipyridamole	Cath >70%	78	82
2003	Ishida *et al*^55^	104	0	Dipyridamole	Cath >70%	84	82
2003	Kinoshita *et al*^57^	27		Dipyridamole	Cath >75%	55	77
2003	Nagel *et al*^59^	90	6	Adenosine	Cath >75%	88	90
2002	Ibrahim *et al*^53^	25		Adenosine	QCA >75%	69	89
2001	Schwitter *et al*^68^	48	1	Dipyridamole	QCA >50%	85	94
2000	Al-Saadi *et al*^49^	40	6	Dipyridamole	Cath >75%	90	83

CMR, cardiovascular magnetic resonance.

**Table 4:** Appropriate indications for the use of CMR^142^*

Detection of CAD: Symptomatic—evaluation of chest pain syndrome (use of vasodilator perfusion CMR or dobutamine stress function CMR)
Intermediate pre-test probability of CAD
ECG uninterpretable OR unable to exercise
Detection of CAD: Symptomatic—evaluation of intracardiac structures (use of MR coronary angiography)
Evaluation of suspected coronary anomalies
Risk assessment with prior test results (use of vasodilator perfusion CMR or dobutamine stress function CMR)
Coronary angiography (catheterisation or CT)
Stenosis of unclear significance
Structure and Function—evaluation of ventricular and valvular function
Procedures may include LV/RV mass and volumes, MR angiography, quantification of valvular disease, and delayed contrast enhancement
Assessment of complex congenital heart disease including anomalies of coronary circulation, great vessels, and cardiac chambers and valves
Procedures may include LV/RV mass and volumes, MR angiography, quantification of valvular disease, and contrast enhancement
Evaluation of LV function following myocardial infarction OR in heart failure patients
Patients with technically limited images from echocardiogram
Quantification of LV function
Discordant information that is clinically significant from prior tests
Evaluation of specific cardiomyopathies (infiltrative (amyloid, sarcoid), HCM, or due to cardiotoxic therapies)
Use of delayed enhancement
Characterisation of native and prosthetic cardiac valves—including planimetry of stenotic disease and quantification of regurgitant disease
Patients with technically limited images from echocardiogram or TEE</item></item-list>
Evaluation for arrhythmogenic right ventricular cardiomyopathy (ARVC)
Patients presenting with syncope or ventricular arrhythmia
Evaluation of myocarditis or myocardial infarction with normal coronary arteries
Positive cardiac enzymes without obstructive atherosclerosis on angiography
Structure and Function—evaluation of intracardiac and extracardiac structures
Evaluation of cardiac mass (suspected tumour or thrombus)
Use of contrast for perfusion and enhancement
Evaluation of pericardial conditions (pericardial mass, constrictive pericarditis)
Evaluation for aortic dissection
Evaluation of pulmonary veins prior to radiofrequency ablation for atrial fibrillation
Left atrial and pulmonary venous anatomy including dimensions of veins for mapping purposes
Detection of myocardial scar and viability—evaluation of myocardial scar (use of late gadolinium enhancement)
To determine the location and extent of myocardial necrosis including “no reflow” regions
Post acute myocardial infarction
To determine viability prior to revascularisation
Establish likelihood of recovery of function with revascularisation (PCI or CABG) or medical therapy
To determine viability prior to revascularisation
Viability assessment by SPECT or dobutamine echo has provided “equivocal or indeterminate” results

*adapted from ACCF/ACR/SCCT/SCMR/ASNC/NASCI/SCAI/SIR 2006 appropriateness criteria for cardiac computed tomography and cardiac magnetic resonance imaging. *J Am Coll Cardiol* 2006;**48**:1475–97.
